# The Meeting of the American Medical Association

**Published:** 1894-07

**Authors:** 


### *EDITORIAL.*

---

The office of THE JOURNAL is in room 330 Equitable Building

Address all communications, and make all remittances payable to THE ATLANTA MEDICAL AND SURGICAL JOURNAL, Atlanta, Ga.

Articles for publication should reach this office not later than the 15th instant.

The editors are not responsible for views expressed by contributors.

---

### THE MEETING OF THE AMERICAN MEDICAL ASSOCIATION.

The forty-fifth annual meeting of the Association was held in San Francisco, June 5th, 6th, 7th and 8th, under the Presidency of Dr. Jas. F. Hibberd, of Indiana. About six hundred members were in attendance. In the President's address he took occasion to advocate the establishment of a Department of Public Health, with a Secretary of Public Health in the President's cabinet. His studies, however, led him to believe that this would not be accomplished within the remainder of the nineteenth century. Dr. Hibberd also spoke with feeling against the late illustration of public economy which seeks to reduce the appropriation for the Surgeon-General's office in Washington. For twelve years the appropriation was $10,000, but the last Congress reduced it to $7,000, and now it is proposed to reduce it still further. Upon this matter the Association took action and telegraphed to Washington its protest against the reduction. The revision of the Constitution and of the Code of Ethics were matters which engaged the attention of the Association. Reports were made by committees appointed at the last meeting of the Association recommending the revision of both the Constitution and the Code along certain lines. Minority reports were made also, advising no interference. After a protracted and somewhat heated discussion the whole matter was disposed of by being laid upon the table indefinitely, and thus the Constitution and the Code remain *in statu quo.* A resolution was adopted recommending the passage of laws in the States for medical examining boards.

The Address in Medicine was delivered by Dr. C. H. Hughes, of St. Louis, who chose for his subject, "The Nervous System in Disease, and the Practice of Medicine from a Neurological Standpoint."

The next meeting of the Association will be held in Baltimore, beginning the first Tuesday in May, 1895. Following are the officers for the current year: President, Dr. Donald McLean, of Michigan; Vice-President, Dr. T. C. Loving, of Ohio; Treasurer, Dr. S. P. Newman, of Illinois; Secretary, Dr. William B. Atkinson, of Pennsylvania; Assistant Secretary, Dr. Geo. H. Rohe, of Maryland; Editor of the Association *Journal*, Dr. J. B. Hamilton, of Illinois.

